# The Effect of Different Thiamethoxam Concentrations on *Riptortus pedestris* Development and Fecundity

**DOI:** 10.3390/toxics12070460

**Published:** 2024-06-26

**Authors:** Zijie Wang, Song Wang, Lixia Li, Lei Chen, Yu Gao, Ming Yuan, Yueying Wang, Shusen Shi

**Affiliations:** 1 College of Plant Protection, Jilin Agricultural University, Changchun 130118, China; wangzijie970102@163.com (Z.W.); 18308657411@163.com (L.L.); sss-63@263.net (S.S.); 2 Jiamusi Branch of Heilongjiang Academy of Agricultural Sciences, Jiamusi 154007, China; 3 Key Laboratory of Soybean Disease and Pest Control, Ministry of Agriculture and Rural Affairs, Changchun 130118, China; 4 Qiqihar Branch of Heilongjiang Academy of Agricultural Sciences, Qiqihar 161006, China; 5 Suzhou Academy of Agricultural Sciences, Suzhou 234099, China

**Keywords:** life table, *Riptortus pedestris*, thiamethoxam, population parameters, soybean

## Abstract

The stink bug, *Riptortus pedestris* (Fabricius) (Hemiptera: Alydidae), is a highly destructive pest that significantly damages legume crops in East and South Asia. Neonicotinoid insecticides containing thiamethoxam are widely used to control *R. pedestris* in soybean fields. However, the current knowledge on the impact of different thiamethoxam concentrations on *R. pedestris* growth and reproduction is lacking and insufficient. The present study investigated the effects of thiamethoxam on the biological traits of *R. pedestris* after treatment with LC_10_ (19.8 mg/L), LC_20_ (31.6 mg/L), LC_30_ (44.2 mg/L), LC_40_ (58.9 mg/L), and LC_50_ (77.0 mg/L) concentrations. These five thiamethoxam concentrations (LC_10_~LC_50_) reduced adult longevity and fecundity in the F_1_ generation females. Thiamethoxam treatment also significantly decreased the population trend index, intrinsic rate of increase, net reproductive rate, gross reproductive rate, and finite rate of increase and increased the mean generation time. These results show that thiamethoxam hinders and suppresses the development and growth of the F_1_ population of *R. pedestris*. Thiamethoxam is recommended for spray control during peak adult emergence, as it not only has a controlling effect on the parental generation but also a negative impact on the F_1_ generations.

## 1. Introduction

The global population is projected to reach 9 billion by 2050; with its continuous growth, it is important to consider how we can achieve food security [1]. Consequently, increasing crop yield has become a global scientific concern [2,3]. Soybean (*Glycine max* (L.) Merr.) is an important grain and oil crop native to China [4]. Soybean crop production is at risk due to the presence of harmful organisms, including insects, weeds, and pathogens [5,6,7]. Many studies have shown that insect pests are the most important factors limiting soybean yield worldwide [5,8,9]. In recent years, the soybean plants of a large area in the Huang-Huai-Hai region of China have presented stay-green syndrome (known as the “Zhengqing” problem in Chinese), with delayed leaf senescence (stay-green), flat pods, and aborted seeds, causing significant economic losses [10,11,12]. At least three types of causal factors associated with stay-green syndrome have been discovered to date: the bean stinkbug *Riptortus pedestris* (Fabricius) (Hemiptera: Alydidae), which pierces and sucks soybean pods at the R_3_–R_5_ stages (from the beginning of pod development to the beginning of seed development) [13,14]; the novel soybean stay-green-associated geminivirus (SoSGV) [15,16,17]; and the virus-transmitting leafhopper, *Orosius orientalis* (Matsumura) (Hemiptera: Cicadellidae) [18,19].

*R. pedestris* is a devastating pest affecting legume crops in East and South Asia [20,21] that is known to attack over 30 plant species of about 13 families and prefers legume crops, such as soybean, mung bean (*Vigna radiata* L.), and pea (*Pisum sativum* L.) [22,23]. Although sustainable pest control methods are being developed [24,25,26], the most efficient measure remains spraying chemical insecticides from the flowering to the pod-filling stage [27,28]. However, the extensive use of insecticides could rapidly increase insecticide resistance [29]. Some insecticides are present at sublethal concentrations after application [30,31]. Sublethal insecticide concentrations, which are insufficient for killing insects, can affect population growth parameters, insect feeding behavior, and nutrient content [32,33]. Previous studies have shown that insecticides, including those containing thiamethoxam (CAS: 153719-23-4), can be used to control *R. pedestris* [28,34]. However, the impact of different thiamethoxam concentrations on *R. pedestris* development and reproduction remains unclear [34]. Therefore, evaluating the concentration effects and overall impact of thiamethoxam on the target pests will aid in scientifically assessing the efficacy of thiamethoxam against *R. pedestris* in field conditions. The lack of knowledge on the sublethal effects of thiamethoxam poses a major challenge in *R. pedestris* management. Our aim was to investigate the potential effects of different thiamethoxam concentrations on *R. pedestris* and to provide a guide on effective insecticide uses in the field. The development parameters, including the developmental duration, pre-oviposition period, longevity, larval and adult survival rate, and daily egg production of individual females of *R. pedestris*, were measured using five thiamethoxam concentrations under laboratory conditions. The population parameters, including the survival rate, fecundity, life expectancy, and reproduction value of *R. pedestris*, were calculated using the age–stage, two-sex life table.

## 2. Materials and Methods

### 2.1. Insects and Soybean Plants

The *R. pedestris* adults were originally collected from soybean fields in Guizhou Province, China (26°30′15″ N, 106°39′19″ E). The adults used in the bioassays were obtained after more than ten generations of continuous laboratory rearing, according to the method described by Tian et al. (2022), at 25 ± 1 °C, 80 ± 5% R.H., and a photoperiod of 16–8 h (light–dark) [35]. Soybean seeds (cultivar “Jinong 38”) were planted in a greenhouse according to the method described by Gao et al. (2022) [23] and used as feed for *R. pedestris*.

### 2.2. Toxicity Bioassay

The thiamethoxam formulation (25%, water-dispersible granules) was purchased from Yuelian Chemical Co., Ltd. (Shanghai, China), and stored in a refrigerator (4 ± 2 °C). Six thiamethoxam concentrations (33.0, 67.0, 100.0, 133.0, 167.0, and 200.0 mg/L) were prepared via serial dilution in distilled water. Thirty-five-day-old fresh soybean pods of uniform size were selected [34], thoroughly washed with clean water, and dried at room temperature. These whole pods were immersed in the serially diluted thiamethoxam solutions for 20 s and then dried at room temperature. A single pod was then released into a centrifuge tube (50 mL) [35] (Figure S1). After 24 h of starvation, five female and five male 3-day-old adults were placed in each centrifuge tube. The pods immersed only in distilled water were used as the control treatment. Three replicates were maintained for each treatment. The number of dead *R. pedestris* was counted after 24 h. Insect mortality was assessed under a stereomicroscope (SZ-61, Olympus Corporation, Tokyo, Japan) and confirmed by the absence of an obvious reaction when the antennae, head, abdomen, legs, and other sensitive parts were lightly touched with a writing brush [34]; the mortality in all control treatments was below 5%.

### 2.3. Effects of Thiamethoxam on the F_1_ Generation of R. pedestris

*R. pedestris* adults were collected and treated with LC_10_, LC_20_, LC_30_, LC_40_, and LD_50_ concentrations of thiamethoxam (19.8 mg/L, 31.6 mg/L, 44.2 mg/L, 58.9 mg/L, and 77.0 mg/L, see Section 3.1—Results), and the samples treated with water were the control group. All exposed samples were considered the parental generation. Soybean seeds of a similar size were selected and soaked in water until soft [34], washed thoroughly with clean water, and dried at room temperature. The whole seeds were dipped in the serially diluted solutions of thiamethoxam (LC_10_, LC_20_, LC_30_, LC_40_, and LC_50_) for 20 s and then dried at room temperature. After 24 h, the live samples of each treatment were reared separately in a single cage (30 cm × 30 cm). The insect cages were placed in an artificial climate room with a temperature of 25 ± 1 °C, 80 ± 5% relative humidity, and a 16–8 h (light–dark) photoperiod [35].

Following the thiamethoxam treatment of the *R. pedestris* parental generation, 120 healthy eggs were collected and transferred to a Petri dish covered with wet filter paper and incubated in an artificial climate chamber (GXZ-380B, Jiangnan Instrument Factory, Ningbo, China). The newly hatched F_1_ generation nymphs were reared in a single centrifuge tube (50 mL) (Figure S1) under the same environmental conditions as above. The individual mortality at each stage was observed and recorded daily. The number of eggs laid was recorded daily until the death of adult females and males. The development time, adult longevity, and daily egg production of individual females of the F_1_ generation under each treatment were recorded daily.

### 2.4. Life Table Parameters

The life table parameters were calculated following the age–stage, two-sex life table procedure (the TWOSEX–MSChart program) using the above-recorded data [36]. The population parameters are calculated using Equations (1) and (2), where *k* represents the number of life stages [37,38,39,40,41].

(1) $$l_{x}=\sum_{j=1}^{k}s_{xj}$$

(2) $$m_{x}=\frac{\sum_{j=1}^{k}s_{xj}f_{xj}}{\sum_{j=1}^{k}s_{xj}}$$

The net reproductive rate (*R*_0_), intrinsic rate of increase (*r*), finite rate of increase (*λ*), mean generation time (*T*), age–stage life expectancy (*e_xj_*), and age–stage reproductive value (*v_xj_*) were calculated using Equations (3)–(8), respectively.

(3) $$R_{0}=\sum_{x=0}^{\infty }l_{x}m_{x}$$

(4) $$\sum_{x=0}^{\infty }e^{-r(x+1)}l_{x}m_{x}=1$$

(5) $$\lambda =e^{r}$$

(6) $$T=\frac{\ln(R_{0})}{r}$$

(7) $$e_{xj}=\sum_{i=x}^{n}\sum_{j=y}^{m}s\prime_{ij}$$

(8) $$v_{xj}=\frac{e^{r(x+1)}}{s_{xj}}\sum_{i=x}^{\infty }e^{-r(i+1)}\sum_{y=j}^{k}s\prime_{iy}f_{iy}$$

The population trend index is expressed as Equation (9) [23], where *I* is the population trend index; *N*_1_ and *N*_0_ are the number of F_1_ and parental generations of *R*. *pedestris*, respectively; *S_E_*, *S*_1_, *S*_2_, *S*_3_, *S*_4_, and *S*_5_ are the *R*. *pedestris* survival rates of eggs and the 1st-, 2nd-, 3rd-, 4th-, and 5th-instar nymphs, respectively; *S_A_* is the survival rate of adults; *F* is the average number of eggs laid per female; and *P_♀_* is the proportion of female adults. *I* > 1 indicates that the next-generation population is higher than the previous one; *I* < 1 means that the next generation will be smaller than the previous one [35].

*I* = *N*_1_/*N*_0_ = *S_E_* × *S*_1_ × *S*_2_ × *S*_3_ × *S*_4_ × *S*_5_ × *S_A_* × *F* × *P_♀_* (9)

### 2.5. Statistical Analyses

The values, slopes, and estimated values of the LC_10_, LC_20_, LC_30_, LC_40_, and LC_50_ (semilethal concentrations) were analyzed via the probability model using the Data Processing System (DPS) software (version 13.5, Hangzhou Ruifeng Information Technology Co., Ltd., Hangzhou, China; website: http://www.dpsw.cn/ (accessed on 22 June 2024) [42]. Significant differences between the treatments in the developmental duration and the life table parameters were determined via a one-way analysis of variance followed by Tukey’s multiple comparisons test. Significant differences between the longevity of females and males were determined via the *t*-test. The SigmaPlot software (version 12.5, Systat Software Inc., San Jose, CA, USA) was used to generate the figures.

## 3. Results

### 3.1. Toxicity Bioassay of Thiamethoxam

The regression equation applied using the DPS software was *y* = 2.18*x* + 7.42 (*χ*^2^ = 0.9007, *p* = 0.9245, *R* = 0.9843) (Table 1). The LC_10_, LC_20_, LC_30_, LC_40_, and LC_50_ of thiamethoxam for *R. pedestris* adults were 19.8 mg/L, 31.6 mg/L, 44.2 mg/L, 58.9 mg/L, and 77.0 mg/L, respectively, after 24 h. The 95% confidence limits are 7.8~31.5, 15.9~44.9, 26.4~58.6, 40.1~74.3, and 58.0~94.8, respectively. These concentrations of thiamethoxam solutions were then used as the treated concentrations for the follow-up experiments.

### 3.2. Effects of Thiamethoxam on the F_1_ Generation of R. pedestris

Thiamethoxam at different concentrations (LC_10_, LC_20_, LC_30_, LC_40_, and LC_50_) significantly influenced the duration of each nymphal developmental stage, adult longevity, adult preoviposition period (APOP), total preoviposition period (TPOP), and population trend index (*I*) of the *R. pedestris* F_1_ generation (Table 1 and Table S1). For eggs, the second-, third-, fourth-, and fifth-instar nymphs, the nymph period, and the total pre-adult stage, increasing thiamethoxam concentrations prolonged the developmental duration. These thiamethoxam treatment values were higher than those of the control treatment (*F_egg_* = 107.96, *p* < 0.0001; *F2nd _instar nymph_* = 19.16, *p* < 0.0001; *F3rd _instar nymph_* = 28.59, *p* < 0.0001; *F4th _instar nymph_* = 39.59, *p* < 0.0001; *F5th _instar nymph_* = 82.90, *p* < 0.0001; *F_nymph period_* = 852.84, *p* < 0.0001; and *F_total pre-adult stage_* = 106.15, *p* < 0.0001). The developmental duration of first-instar nymphs under the LC_30_ treatment was significantly longer than that under other treatments (*F1st _instar nymph_* = 11.92, *p* = 0.0003). The longevity of both female and male adults decreased significantly with increasing thiamethoxam concentrations (*F_female adult_* = 7.268, *p* = 0.0024; *F_male adult_* = 8.626, *p* = 0.011) and was the shortest under the LC_30_ treatment. There was no significant difference between males and females at the same concentration (control: *p* = 0.5015; LC_10_: *p* = 0.4778; LC_20_: *p* = 0.1973; LC_30_: *p* = 0.119; LC_40_: *p* = 0.4327; and LC_50_: *p* = 0.3625). The APOP of the LC_10_ treatment was significantly longer than that of the control treatment (*F* = 14.44, *p* < 0.0001). The LC_40_ and LC_50_ treatments prolonged the TPOP (43.40 ± 0.29 days and 44.00 ± 0.53 days) when compared with the control treatment (*F* = 40.35, *p* < 0.0001).

The population parameters of the *R. pedestris* F_1_ generation are listed in Table 2. The intrinsic rate of increase (*r*), net reproductive rate (*R*_0_), finite rate of increase (*λ*), and gross reproductive rate (GRR) decreased with increasing thiamethoxam concentrations (*F_rm_* = 3.917, *p* = 0.0245; *F_λ_* = 4.068, *p* = 0.0216; *F_R0_* = 6.555, *p* = 0.0037; and *F_GRR_* = 5.835, *p* = 0.0058); these values were significantly lower than those of the control treatment. The LC_40_ treatment demonstrated a higher mean generation time (*T*) (53.6654 ± 2.2354 days) than the control (43.9912 ± 3.4052 days). For the gross reproductive rate, there was no significant difference among the LC_10_, LC_20_, LC_30_, LC_40_, and LC_50_ treatments (*F* = 6.422, *p* = 0.004). This observation indicated that five thiamethoxam concentrations caused different degrees of adverse effects on the population parameters of *R. pedestris*.

### 3.3. Age–Stage-Specific Survival Rate (S_xj_)

**Figure 1:**
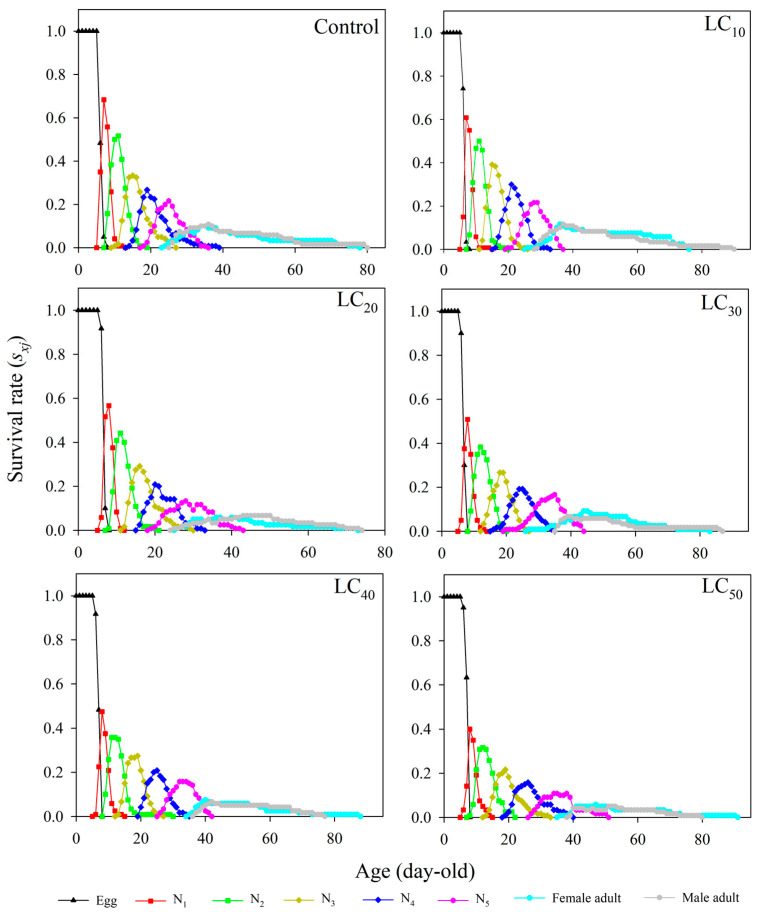
Age–stage-specific survival rate (*s_xj_*) of *Riptortus pedestris* at different thiamethoxam concentration treatments. Note: N_1_, N_2_, N_3_, N_4_, and N_5_ represent first-, second-, third-, fourth-, and fifth-instar nymphs, respectively.

*R. pedestris* completed generational development under the five tested thiamethoxam concentrations (LC_10_~LC_50_). The age–stage-specific survival rates of the first-, second-, third-, fourth-, and fifth-instar nymphs and adult females and males in the LC_10_~LC_50_ treatments were shorter than those in the control treatment (Figure 1). The developmental duration of females was shorter than those of males in the control, LC_10_, LC_20_, and LC_30_ treatments. However, the developmental duration of males was shorter than that of females at LC_40_ and LC_50_ concentrations. These observations suggest that adult males may be more sensitive to high thiamethoxam concentrations than females and therefore more susceptible to lethality. The survival rates at each developmental stage initially increased and then decreased as the concentration increased from LC_10_ to LC_50_. The survival rates were the highest in the control treatment and the lowest in the LC_50_ treatment; the survival rate values were 68.33%, 51.67%, 33.33%, 26.67%, and 21.67% under the control treatment and 40.00%, 31.67%, 21.67%, 15.83%, and 10.83% under the LC_50_ treatment for the first-, second-, third-, fourth-, and fifth-instar nymphs, respectively. An apparent overlap in the age–stage survival curves was observed due to the individual differences in mortality under different thiamethoxam concentrations. These results indicate that thiamethoxam in the five concentrations negatively influences the survival rate and duration of the F_1_ generation of *R. pedestris*, with males being more affected than females.

### 3.4. Age-Specific Survivability and Age–Stage-Specific Fecundity

**Figure 2:**
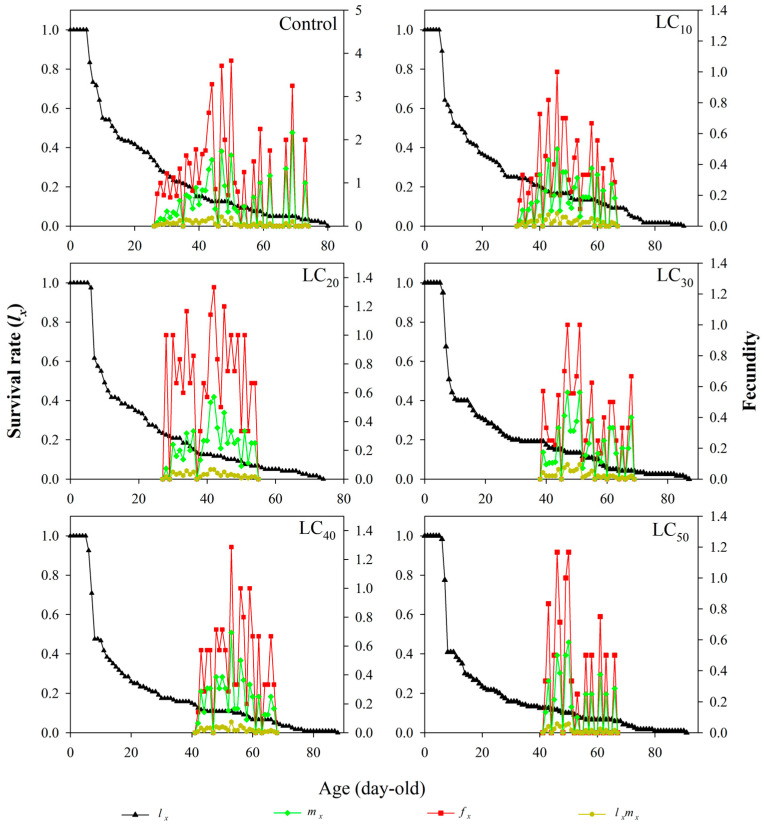
Age-specific survival rate (*l_x_*), age-specific fecundity (*m_x_*), age–stage-specific fecundity (*f_x_*), and age-specific maternity (*l_x_m_x_*) of *R. pedestris* at different thiamethoxam concentration treatments.

The age-specific survival rate (*l_x_*) decreased sharply with the increasing age of the test insects within each thiamethoxam concentration tested from LC_10_ to LC_50_ (Figure 2). The age–stage-specific fecundity (*m_x_*) ceased (no further oviposition) at ages (day) of 73, 66, 56, 68, 67, and 68 for the CK, LC_10_, LC_20_, LC_30_, LC_40_, and LC_50_ treatments, respectively. Moreover, the highest age-specific fecundities were recorded: 2.1667 (69 day, CK), 0.5000 (46 day, LC_10_), 0.5714 (42 day, LC_20_), 0.5625 (47/51 day, LC_30_), 0.6923 (53 day, LC_40_), and 0.5833 (50 day, LC_50_) eggs. The peak values of age-specific maternity (*l_x_m_x_*) were 0.2167 at 47 day, 0.0833 at 46 day, 0.0667 at 41~42 day, 0.075 at 47/51 day, 0.075 at 53 day, and 0.0583 at 46/50 day for the CK, LC_10_, LC_20_, LC_30_, LC_40_, and LC_50_ treatments, respectively.

### 3.5. Age–Stage-Specific Life Expectancy

**Figure 3:**
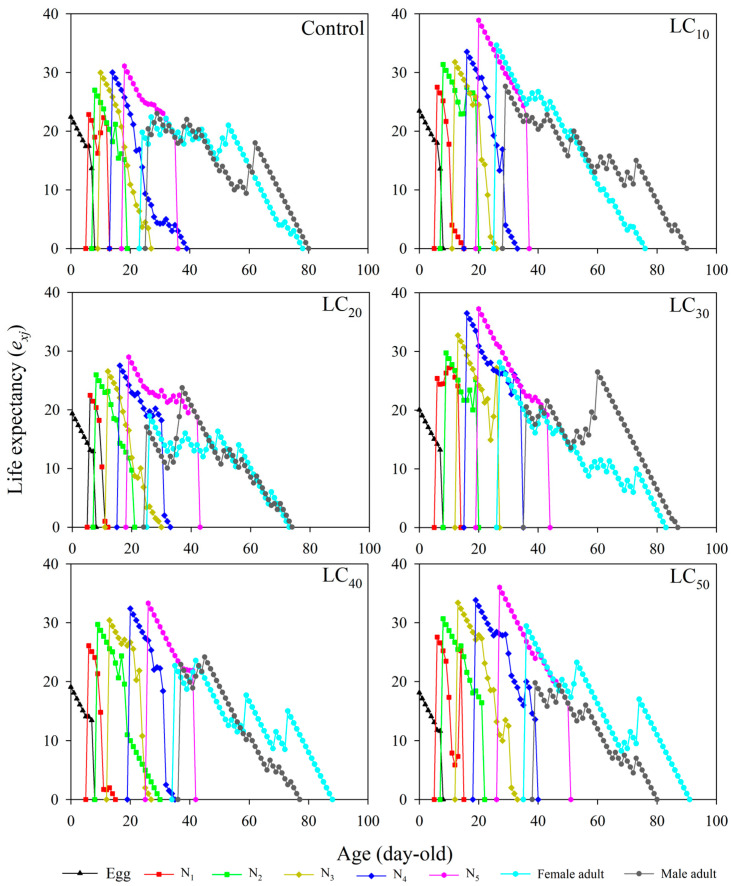
Life expectancy (*e_xj_*) of *R. pedestris* at different thiamethoxam concentration treatments. Note: N_1_, N_2_, N_3_, N_4_, and N_5_ represent first-, second-, third-, fourth-, and fifth-instar nymphs, respectively.

The life expectancy (*e_xj_*) value of a given stage decreased with increasing concentrations and, for the same thiamethoxam concentration, decreased with increasing age (Figure 3). The highest life expectancy of female adults was 34.6429 (26 day) under LC_10_, and the lowest was 18.9529 under LC_20_ (26 day) (Figure 3). Similarly, the highest life expectancy of male adults was 27.6496 under LC_10_ (29 day), and the lowest life expectancy was 19.7733 under LC_20_ (39 day).

### 3.6. Age–Stage-Specific Reproductive Values

**Figure 4:**
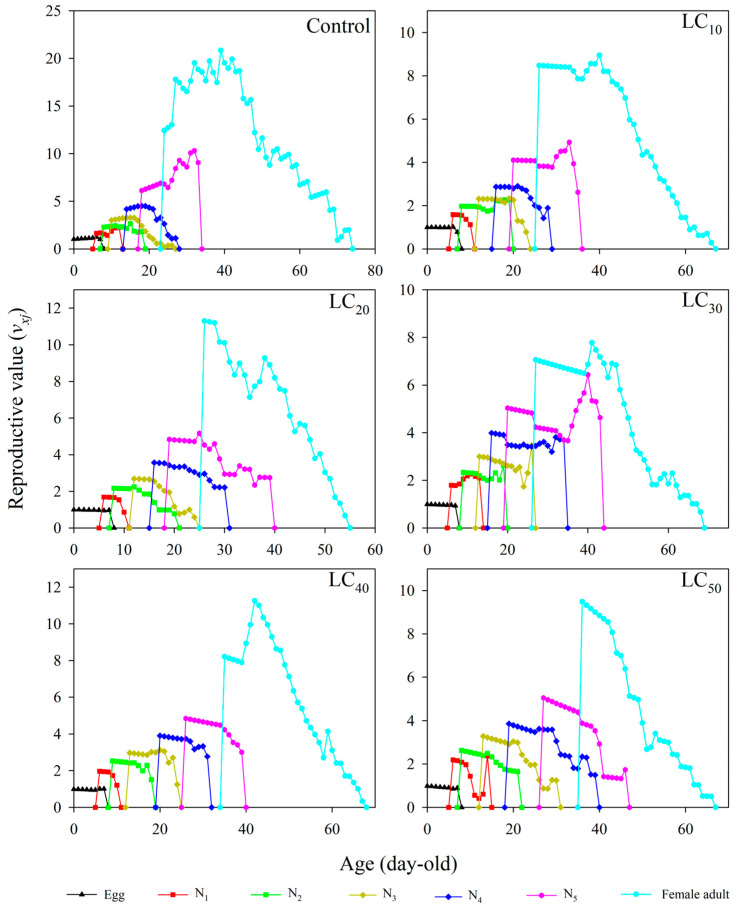
Reproductive value (*v_xj_*) of *R. pedestris* at different thiamethoxam concentration treatments. Note: N_1_, N_2_, N_3_, N_4_, and N_5_ represent first-, second-, third-, fourth-, and fifth-instar nymphs, respectively.

The age–stage-specific reproductive values (*v_xj_*) gradually increased with increasing age (*x*) and stage (*j*) (Figure 4). The first *v_xj_* values from female adults were detected at 24 day (CK), 26 day (LC_10_), 26 day (LC_20_), 27 day (LC_30_), 35 day (LC_40_), and 36 day (LC_50_). The highest *v_xj_* values of female adults occurred at 39 day (20.8419 d^−1^), 40 day (8.9443 d^−1^), 26 day (11.3011 d^−1^), 41 day (7.7804 d^−1^), 42 day (11.2579 d^−1^), and 36 day (9.4834 d^−1^) of age.

## 4. Discussion

In this study, the logarithm of the mortality rate of the test insects versus the concentration is in the form of a cumulative times curve. Thus, the DPS software was used to transform the relationship into a straight line to facilitate the subsequent calculations. The life table, which is an effective method for studying the population dynamics of arthropods [43], is used to more accurately investigate the potential effects of different thiamethoxam concentrations on *R. pedestris*. The age–stage, two-sex life table overcomes the limitations of traditional life tables by considering the age differentiation of insects and incorporating all individuals within the population. This approach facilitates an accurate depiction of age stratification, predicts population growth trends, and provides a correct analysis of parameters, including the reproduction rate [38,39]. Utilizing this technique offers an effective means to access the impact of external environmental factors on the population dynamics of *R. pedestris*, such as the temperatures, host plants, and insecticides [32,35]. In our study, plotting the *s_xj_*, *l_x_*, *m_x_*, *l_x_m_x_*, and *e_xj_* curves revealed the adverse effects of thiamethoxam at LC_10_~LC_50_ concentrations on the F_1_ population growth parameters of *R. pedestris* using the age–stage, two-sex life table. After the LC_10_~LC_50_ treatments of parental adult *R. pedestris*, the population trend index, survival rate, longevity, and fecundity of the F_1_ generation populations of *R. pedestris* significantly decreased. Our results indicated that the F_1_ generation showed a longer developmental period; lower *r_m_*, *λ*, and *R_0_* values; and a longer mean generation time after the parental generation nymphs were treated. The intrinsic rate of increase (*r*) of the LC_10_, LC_20_, LC_30_, LC_40_, and LC_50_ treatments reflected the reproductive capacity of the *R. pedestris* population after thiamethoxam treatment under ideal conditions. Similarly, the effects of thiamethoxam on life table parameters have been reported in several hemipteran insects or their natural enemies, such as *Laodelphax striatellus* (Fallén) (Delphacidae) [44], *Myzus persicae* (Sulzer) (Aphididae) [45], *Sogatella furcifera* (Horváth) (Delphacidae) [46], *Brevicoryne brassicae* (L.) (Aphididae) [47], and *Cyrtorhinus lividipennis* Reuter (Miridae), an important predator of *Nilaparvata lugens* (Stål) (Delphacidae) [48]. These findings highlight that the surviving individuals experience a decline in survival suitability after insecticide treatment [49]. Insecticides at decreased concentrations can seriously affect the population growth of exposed insects [50]. A significant reduction in the population parameters was also observed in *Brevicoryne brassicae* (L.) and *Aphis gossypii* Glover (Aphididae) after exposure to sublethal concentrations [51,52]. Therefore, a systematic description of the effects of thiamethoxam in different concentrations, including changes in developmental stages and fecundity, will contribute to effective thiamethoxam use [53].

Depending on the biological trait studied, either positive or negative effects were observed at the individual (intra-generational) and transgenerational levels [54]. This study demonstrated that thiamethoxam has notable effects on the progeny of the directly exposed individuals, referred to as transgenerational effects. Following parental exposure to the LC_10_~LC_50_ concentrations of thiamethoxam, the fecundity of the progeny decreased compared to the control. Furthermore, the thiamethoxam treatment of the parental generation of adult *R. pedestris* had a more potent adverse effect on the F_1_ generation than it did on the third-instar nymphs [34]. A similar study showed that the LC_30_ concentrations of lambda-cyhalothrin and emamectin benzoate exhibited a transgenerational effect on the third-instar nymphs of *R. pedestris* [32]. Lethal, sublethal, and transgenerational effects of the neonicotinoid insecticides have also been reported on many economic insects, such as *Apolygus lucorum* (Meyer-Dür) (Miridae) [55], *Cryptolaemus montrouzieri* Mulsant (Coccinellidae) [56], *Coccinella septempunctata* L. (Coccinellidae) [57], and *N. lugens* (Stål) [58]. Moreover, the LD_20_ concentration of imidacloprid and dinotefuran significantly benefited *N. lugens* (Stål) [59]. These pests were likely exposed to relatively low concentrations of insecticides under real field conditions. The side effects or sublethal toxicity of insecticides are nearly as severe as their lethal toxicity against insects [60]. Assessing insecticide effects on insect development and reproduction, especially at the multigenerational level, provides data for understanding the potential relationship between insecticide use and insect occurrence [61,62,63]. Our results can provide a reference for further improving the evaluation of thiamethoxam’s field control effectiveness. Thiamethoxam and other neonicotinoid insecticides are used for controlling agricultural and forestry pests worldwide [64,65]. Thiamethoxam has been demonstrated as an excellent organic pesticide [66]; however, the mechanism of action of thiamethoxam against the parental and F_1_ generations of *R. pedestris* requires further investigation.

Applying chemical pesticides is still the most common pest control measure [6]. The selection pressure of insecticides containing thiamethoxam is high in soybean fields in Northern China, which are sprayed two or four times during the flowering stage [10,13]. In addition, thiamethoxam is applied through seed coating in soybean and does not affect seed germination [67]. Thiamethoxam and its main metabolite (clothianidin) exhibited a less notable effect on nontarget piercing–sucking herbivores and the earthworm *Eisenia fetida* (Oligochaeta: Lumbricidae) [68,69]. Thiamethoxam is a degradable pesticide that has a half-life period of 7.1–92.3 days in European field soil [70]. To date, no surveillance data indicate the increased moderate-to-high levels of thiamethoxam resistance in the field populations of *R. pedestris*. Therefore, the risk of *R. pedestris* developing resistance to thiamethoxam warrants further study. In addition to the acute effects that typically occur at high concentrations, the sublethal and transgenerational effects in pest populations can occur over time. Thus, field studies are necessary to conclude the potential role of this insecticide in suppressing pest populations. Although these findings need to be validated under real cropping conditions, along with the acute effects that typically occur at high dosages, it is expected that sublethal and transgenerational effects may occur in pest populations over time due to the current pesticide applications. Our data will help understand the effects of thiamethoxam on the lifecycle of *R. pedestris* and formulate long-term, effective control strategies to mitigate insecticide resistance.

## 5. Conclusions

We conducted an indoor study to investigate the impact of various thiamethoxam concentrations on the biological characteristics of the parental and F_1_ generations of *R. pedestris*. The five concentrations of thiamethoxam significantly decreased the survival rate and fecundity of the F_1_ generation of *R. pedestris*. Thiamethoxam treatment also significantly reduced the population trend index, intrinsic rate of increase, net reproductive rate, and finite rate of increase and increased the generation time, which suggests that the field control of the parental generation of adult *R. pedestris* using thiamethoxam still has a sustained controlling effect on their F_1_ generation population. Thiamethoxam is recommended for spray control during the adult peak emergence. The results can provide a theoretical basis for the sustainable management of *R. pedestris* and the scientific and effective use of thiamethoxam.

## Figures and Tables

**Table 1:** The developmental durations, longevity, APOP, and TPOP of the F_1_ generation of *R. pedestris* at LC_10_, LC_20_, LC_30_, LC_40_, and LC_50_ concentrations of thiamethoxam.

Treatments	Development Duration of Developmental Stages of F_1_ Generation (Days)										APOP (Days)	TPOP (Days)
	Egg	First-Instar Nymph	Second-Instar Nymph	Third-Instar Nymph	Fourth-Instar Nymph	Fifth-Instar Nymph	Nymph Period	Total Pre-Adult Stage	Female Adult	Male Adult		
Control	6.56 ± 0.03 ^e^	2.52 ± 0.06 ^b^	4.49 ± 0.12 ^d^	4.86 ± 0.14 ^b^	4.90 ± 0.11 ^e^	6.97 ± 0.13 ^d^	23.74 ± 0.11 ^d^	29.09 ± 0.33 ^d^	15.09 ± 2.13 ^a^	16.09 ± 2.28 ^a^	3.80 ± 0.22 ^cd^	31.59 ± 0.94 ^c^
LC_10_	6.79 ± 0.03 ^d^	2.52 ± 0.05 ^b^	4.71 ± 0.12 ^d^	5.41 ± 0.11 ^b^	5.93 ± 0.12 ^d^	7.93 ± 0.11 ^c^	26.50 ± 0.10 ^c^	32.76 ± 0.30 ^c^	14.02 ± 2.04 ^a^	15.04 ± 2.12 ^ab^	6.00 ± 0.30 ^a^	38.68 ± 1.00 ^b^
LC_20_	7.00 ± 0.03 ^c^	2.70 ± 0.05 ^b^	4.84 ± 0.17 ^cd^	4.94 ± 0.13 ^b^	6.16 ± 0.24 ^cd^	8.17 ± 0.20 ^c^	26.81 ± 0.16 ^c^	33.12 ± 0.59 ^c^	11.01 ± 1.82 ^a^	13.01 ± 2.00 ^abc^	2.67 ± 0.20 ^d^	33.33 ± 1.19 ^c^
LC_30_	7.19 ± 0.04 ^b^	3.12 ± 0.09 ^a^	5.57 ± 0.13 ^ab^	6.14 ± 0.12 ^a^	7.09 ± 0.20 ^bc^	9.35 ± 0.12 ^b^	31.27 ± 0.13 ^b^	38.26 ± 0.48 ^b^	13.04 ± 1.98 ^ab^	10.03 ± 1.78 ^bc^	4.00 ± 0.34 ^bcd^	42.33 ± 0.77 ^ab^
LC_40_	7.51 ± 0.04 ^a^	2.53 ± 0.06 ^b^	5.46 ± 0.13 ^bc^	6.20 ± 0.09 ^a^	7.21 ± 0.16 ^b^	9.58 ± 0.11 ^b^	30.98 ± 0.11 ^b^	38.58 ± 0.25 ^b^	10.05 ± 1.76 ^ab^	9.06 ± 1.72 ^c^	5.40 ± 0.40 ^ab^	43.40 ± 0.29 ^a^
LC_50_	7.57 ± 0.05 ^a^	2.69 ± 0.09 ^b^	6.24 ± 0.21 ^a^	6.68 ± 0.22 ^a^	8.52 ± 0.30 ^a^	10.93 ± 0.22 ^a^	35.06 ± 0.21 ^a^	41.98 ± 0.66 ^a^	7.12 ± 1.48 ^b^	8.12 ± 1.71 ^c^	5.00 ± 0.40 ^abc^	44.00 ± 0.53 ^a^

Note: Data are mean ± SE in this table. Different lowercase letters indicate significant differences in the same column (Tukey’s multiple comparisons; *p* < 0.05). APOP: adult preoviposition period. TPOP: total preoviposition period.

**Table 2:** The population parameters of F_1_ generation of *R. pedestris* at LC_10_, LC_20_, LC_30_, LC_40_, and LC_50_ concentrations of thiamethoxam.

Treatments	Intrinsic Rate of Increase (*r*) (per Day)	Finite Rate of Increase (*λ*) (per Day)	Net Reproductive Rate (*R*_0_) (per Offspring Individual)	Mean Generation Time (*T*) (Days)	Gross Reproductive Rate (per Female)
Control	0.0236 ± 0.0126 ^a^	1.0239 ± 0.0128 ^a^	2.8250 ± 1.2880 ^a^	43.9912 ± 3.4052 ^bc^	23.3942 ± 11.1263 ^a^
LC_10_	−0.0014 ± 0.0095 ^ab^	0.9986 ± 0.0094 ^ab^	0.9333 ± 0.3796 ^ab^	49.3404 ± 3.8272 ^ab^	5.9912 ± 2.1338 ^b^
LC_20_	−0.0047 ± 0.0151 ^ab^	0.9953 ± 0.0147 ^ab^	0.8250 ± 0.3839 ^ab^	41.2273 ± 3.5944 ^c^	6.6078 ± 3.0136 ^b^
LC_30_	−0.0070 ± 0.0122 ^ab^	0.9931 ± 0.0119 ^ab^	0.7000 ± 0.3264 ^ab^	51.1990 ± 3.9177 ^ab^	6.4627 ± 3.2590 ^b^
LC_40_	−0.0098 ± 0.0098 ^ab^	0.9903 ± 0.0096 ^ab^	0.5917 ± 0.2643 ^b^	53.6654 ± 2.2354 ^a^	6.1293 ± 2.4077 ^b^
LC_50_	−0.0174 ± 0.0135 ^b^	0.9828 ± 0.0131 ^b^	0.4083 ± 0.2178 ^b^	51.5577 ± 2.4927 ^ab^	4.3263 ± 2.3479 ^b^

Note: Mean treatment values followed by different letters are significantly different from the control in the same row (*p* < 0.05). The standard error of the mean (SE) was calculated using a bootstrap technique by resampling 100,000 times. Differences between treatments were assessed using a paired bootstrap test.

## Data Availability

Data are contained within this article and the Supplementary Materials.

## Supplementary Materials

The following supporting information can be downloaded at: https://www.mdpi.com/article/10.3390/toxics12070460/s1, Figure S1: Schematic sketch of the self-made device. Table S1: Effects of different thiamethoxam concentrations on the life table parameters of *Riptortus pedestris*.
