# Supplementary material for: The Effect of Different Thiamethoxam Concentrations on Riptortus pedestris Development and Fecundity
Source: Toxics. 2024 Jun 26;12(7):460. doi: 10.3390/toxics12070460 (PMC11280779; doi:10.3390/toxics12070460)
Supplement: Supplementary file 1 [file toxics-12-00460-s001.zip › Table S1.pdf]

Table S1 Effects of different thiamethoxam concentrations on the life table parameters of *Riptortus pedestris.*

Treatments	Proportion of females (%)	Number of eggs laid per female (eggs)	Estimated fecundity of the next generation (eggs)	Population trend index (*I*)
Control	48.39	22.67	340.00	2.83
LC_{10}	48.28	23.14	324.00	2.70
LC_{20}	45.83	27.82	306.00	2.55
LC_{30}	47.83	21.82	240.00	2.00
LC_{40}	52.63	22.60	226.00	1.88
LC_{50}	46.67	24.14	169.00	1.41
