# Supplementary figures and images for: The Effect of Different Thiamethoxam Concentrations on Riptortus pedestris Development and Fecundity
Source: Toxics. 2024 Jun 26;12(7):460. doi: 10.3390/toxics12070460 (PMC11280779; doi:10.3390/toxics12070460)

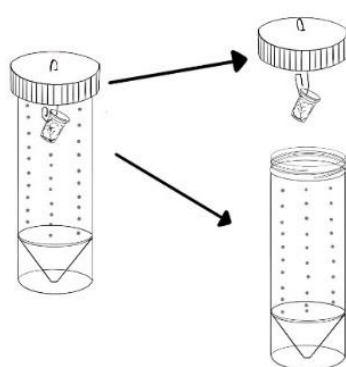

Figure S1 Schematic sketch of the self-made device (Drawn by Mr. Zilong Liu)

Supplement: Supplementary file 1 [file toxics-12-00460-s001.zip › Figure S1.pdf]
